# Cumulative Damage: Cell Death in Posthemorrhagic Hydrocephalus of Prematurity

**DOI:** 10.3390/cells10081911

**Published:** 2021-07-28

**Authors:** Riley Sevensky, Jessie C. Newville, Ho Lam Tang, Shenandoah Robinson, Lauren L. Jantzie

**Affiliations:** 1 Division of Neonatal-Perinatal Medicine, Department of Pediatrics, Johns Hopkins University School of Medicine, Baltimore, MD 21205, USA; rileysevensky@gmail.com (R.S.); newvillejessie@gmail.com (J.C.N.); 2 Division of Pediatric Neurosurgery, Department of Neurosurgery, Johns Hopkins University School of Medicine, Baltimore, MD 21205, USA; holamtang@jhmi.edu (H.L.T.); srobin81@jhmi.edu (S.R.); 3 Department of Neurology and Developmental Medicine, Kennedy Krieger Institute, Baltimore, MD 21205, USA; 4 Department of Neurology, Johns Hopkins University School of Medicine, Baltimore, MD 21205, USA

**Keywords:** cell death, encephalopathy of prematurity, hydrocephalus, preterm birth, neurodevelopment, inflammation, ependyma, glymphatic system, choroid plexus, ferroptosis

## Abstract

Globally, approximately 11% of all infants are born preterm, prior to 37 weeks’ gestation. In these high-risk neonates, encephalopathy of prematurity (EoP) is a major cause of both morbidity and mortality, especially for neonates who are born very preterm (<32 weeks gestation). EoP encompasses numerous types of preterm birth-related brain abnormalities and injuries, and can culminate in a diverse array of neurodevelopmental impairments. Of note, posthemorrhagic hydrocephalus of prematurity (PHHP) can be conceptualized as a severe manifestation of EoP. PHHP impacts the immature neonatal brain at a crucial timepoint during neurodevelopment, and can result in permanent, detrimental consequences to not only cerebrospinal fluid (CSF) dynamics, but also to white and gray matter development. In this review, the relevant literature related to the diverse mechanisms of cell death in the setting of PHHP will be thoroughly discussed. Loss of the epithelial cells of the choroid plexus, ependymal cells and their motile cilia, and cellular structures within the glymphatic system are of particular interest. Greater insights into the injuries, initiating targets, and downstream signaling pathways involved in excess cell death shed light on promising areas for therapeutic intervention. This will bolster current efforts to prevent, mitigate, and reverse the consequential brain remodeling that occurs as a result of hydrocephalus and other components of EoP.

## 1. Introduction

### 1.1. A Broad Introduction to Infantile Hydrocephalus

During infancy, hydrocephalus is characterized by the accumulation of cerebrospinal fluid (CSF) in the brain, progressive macrocephaly, ventriculomegaly, and increased intracranial pressure (ICP) [1,2,3]. Infantile hydrocephalus most often arises in the setting of infection, hemorrhage, trauma, and myelomeningocele, and less frequently from cardiac and genetic defects [1,4,5]. Acquired hydrocephalus commonly requires the surgical insertion of a shunt to drain excess CSF and relieve elevated ICP [6]. While necessary to prevent decline from high ICP, shunt placement early in life is associated with an elevated risk of shunt failure, increased number of shunt revisions over the lifetime, and heightened probability of shunt-related infection, placing these young, vulnerable patients at even greater risk of neurological complications [7,8,9,10,11]. Other possible therapeutic procedures include endoscopic third ventriculostomies, with or without choroid plexus coagulation, which also introduce similar concerns alongside varying reports of efficacy [12,13,14,15].

Affecting nearly 1 of every 1000 live births, hydrocephalus presents a complex variety of both etiology and pathogenesis [16]. Spanning from genetics to hemorrhage to infection to trauma, the initiating factors are incompletely understood and exhaustive, yet often overlapping. Additional conditions, such as chorioamnionitis and preterm birth, also appear to influence evolution of hydrocephalus in infants.

### 1.2. Encephalopathy of Prematurity

Globally, preterm birth is one of the leading causes of neonatal death, with approximately 15 million infants born preterm each year [17,18]. These staggering statistics highlight a realm in which medical and scientific advances are critically needed. Compilation of epidemiological data from the large EPICURE, EPIPAGE, and ELGAN studies spanning Europe and the United States indicates that over a quarter of very preterm infants (<28 weeks’ gestation) ultimately develop a neurological disorder [19,20,21,22]. While preterm infants born later than 28 weeks face lower risk, a significant portion also develop some form of encephalopathy [23]. As such, ongoing research efforts aimed at developing a more thorough understanding of the myriad of neurological consequences affecting preterm neonates is of great significance.

Encephalopathy of prematurity is a broad, overarching classification which encompasses a great multitude of distinct neurological injuries and illnesses that develop during the critical perinatal developmental period [24,25]. While the pathophysiology of encephalopathy of prematurity is complex and multivariate, principal etiological factors include systemic inflammation and hypoxia-ischemia, which have the destructive potential to result in deleterious lifelong neurological consequences [26,27,28].

### 1.3. Chorioamnionitis as a Driver of Dysfunction through the Maternal–Placental–Fetal Axis

Chorioamnionitis (CHORIO) is a major cause of preterm birth [29,30,31,32]. A compelling 40–70% of preterm births are complicated by CHORIO, compared to only 1–13% of full-term births [33,34,35]. Propagated by inflammation through the maternal–placental–fetal axis, chorioamnionitis creates a detrimental microenvironment for the developing nervous system, and can cause significant neurological injury and alteration of neurodevelopmental trajectory.

CHORIO refers to acute intrauterine infection or inflammation (or both) involving the chorioamnionic membranes of the placenta and the umbilical cord during pregnancy [30]. CHORIO can be classified as clinical or histological. Clinical CHORIO is indicated by maternal symptomology of infection (fever, leukocytosis, tachycardia). By contrast, rather than presenting with clinical symptoms, histological CHORIO is defined as pathologically identified inflammation of the chorion, amnion, and placenta, and can present in a variety of complex manners, including neutrophilia [36]. 

CHORIO is considered a common complication of pregnancy, and can have extensive consequences, both for mother and fetus [37]. The intra-amniotic inflammation which characterizes CHORIO has also been intricately linked to induction of encephalopathy of prematurity, a doubled risk of intraventricular hemorrhage (IVH), and a heightened probability of evolution to post-hemorrhagic hydrocephalus of prematurity (PHHP), all of which will be explored further in this review [38,39,40,41].

### 1.4. Posthemorrhagic Hydrocephalus of Prematurity as a Severe Manifestation

Posthemorrhagic hydrocephalus of prematurity is the most prevalent form of hydrocephalus among preterm infants, and refers to the development of hydrocephalus following an intraventricular hemorrhage (IVH) in a preterm neonate [7,42]. In very preterm neonates, underdeveloped cerebral structures and incompletely refined vasculature combine to create an environment which is particularly vulnerable to such insults [7,16]. Up to 20% of premature infants with low birth weight suffer from IVH [43]. Due to the rapid and ongoing neurodevelopment during this sensitive period, hemorrhage and subsequent development of hydrocephalus can have a multitude of consequences [42,44]. 

While the transition from a post-hemorrhagic state to hydrocephalus is still an area of active and robust investigation, hypoxic-ischemic conditions have been implicated in activating a cascade of neuroinflammation that results in bidirectional activation of the neonatal immune system [45,46]. Of note, these factors have been implicated in encephalopathy of prematurity [24], suggesting PHHP may have mechanistic overlap with encephalopathy of prematurity. Children with encephalopathy from very preterm birth are at high risk for social and emotional challenges, in addition to cognitive difficulties [47]. Preterm infants who suffer from PHHP are at even greater risk of lifelong adverse impact on quality of life due to the unpredictable durability of hydrocephalus treatment, and additional medical complexity [48]. While the cumulative challenges of living with PHHP are daunting, greater recognition by the medical community will ideally allow families to cope better [49]. The recent struggles with rationing of medical care have only highlighted the need for more rigorous research to dissect the fundamental pathogenesis of hydrocephalus, particularly PHHP. 

### 1.5. Other Forms of Hydrocephalus

In addition to PHHP, there are other diverse forms of hydrocephalus prevalent in neonates and young children. Primary congenital hydrocephalus is highly dependent on a combination of genetic factors and abnormal structural development in the central nervous system (CNS) [50]. One example of such a cerebral malformation is aqueductal stenosis, in which CSF flow and drainage is inhibited by occlusion of the Aqueduct of Sylvius [51,52]. Post-infectious hydrocephalus (PIH) refers to hydrocephalic progression triggered by infection or severe inflammation in the brain tissue [16]. Most commonly, postnatal contraction of meningitis can introduce external pathogens to the developing nervous system and induce PIH [53,54,55]. Inflammation could also be conferred prior to birth or during delivery, such as via TORCH infections, which initiate immune cell activation along the maternal–placental–fetal axis, and can result in PIH [56,57,58]. While less common in Europe and North America, infantile PIH represents the most common cause of mortality related to hydrocephalus in both Asia and Africa [5,59,60]. Further, post-traumatic hydrocephalus (PTH) is an additional form of acquired childhood hydrocephalus initiated by traumatic brain injury (TBI) [61,62].

### 1.6. A Focus on Cell Death

**Figure 1:**
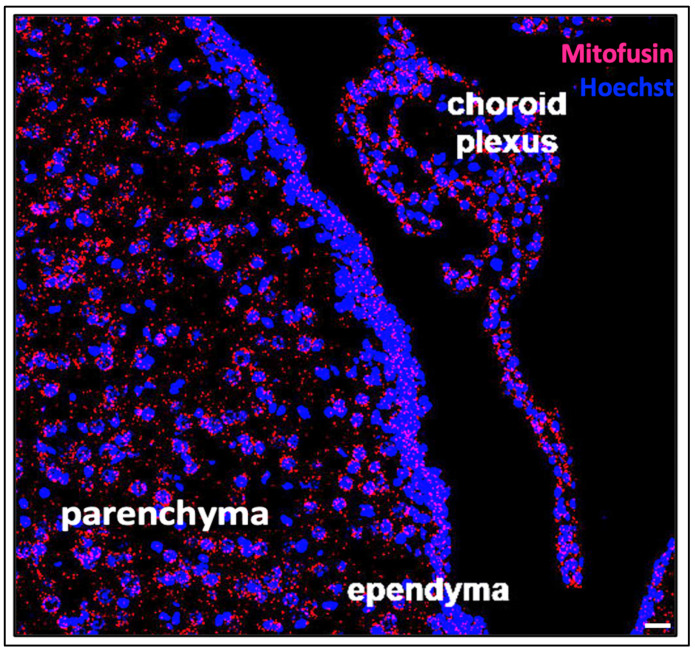
Mitofusin mRNA expression in the ventricular system and the components of cerebral CSF dynamics. Cells in the ependyma, choroid plexus and parenchyma have significant functional metabolic requirements, and can therefore be visualized using RNAscope for mitofusin mRNA in mitochondria (red). These components of the ventricular system are vulnerable to apoptotic, necrotic, phagocytic and ferroptotic forms of cell death due to high energy and metabolic demands, which will be explored in this review. Cell nuclei are labelled with Hoechst and in blue. (Scale bar = 20 microns).

Under the umbrella of brain injuries that contribute to encephalopathy of prematurity, the vast majority involve hypoxic, ischemic, and inflammatory conditions [63]. For the newly generated cells of the CNS in various stages of maturation, these noxious conditions often induce severe damage [64]. For the developing CNS as a whole, widespread damage and excess neural cell loss can be detrimental to neurodevelopment [65]. Specifically, excess cell death in the setting of encephalopathy of prematurity carries substantial significance due to the finality and downstream impacts of cell loss, especially in post-mitotic neural cell populations [66,67,68]. Thus, cell death during the perinatal period can precipitate sustained, detrimental, abnormal neurodevelopment [69]. Of note, the evolution of the components of cerebral CSF dynamics directly overlaps with the timing of factors that contribute to PHHP [70,71,72,73,74,75]. These overlapping insults, including systemic inflammation from CHORIO and/or IVH, impact the maturation of neural cells, including the choroid plexus, ependyma, and glymphatic system (Figure 1). The aim of this review is to explore the literature related to cell death occurring in these CSF-related components within the setting of acquired PHHP, to establish a basis for further investigation of related mechanistic pathways and interventional targets. We suggest that a unifying pathophysiology around cell death within the broader classification of perinatal hydrocephalus exists.

## 2. Cell Death in the Choroid Plexus

### 2.1. Cellular Structure of the Choroid Plexus

Suspended within the cerebral ventricles, the choroid plexus is made up of a single layer of cuboidal epithelial cells aligned in a highly organized fashion, surrounding stroma containing clustered fenestrated capillaries [70]. Early in gestation, shortly after neural tube closure, the choroid plexus forms and begins to secrete trophic factors, cytokines, and CSF [70,76]. Indeed, the development and secretory function of the choroid plexus plays an important role in fluid homeostasis and is thus a critical influencing factor throughout brain development [70]. CSF is produced primarily by the choroid plexus, but also by the ependyma lining of the ventricular system [73,77]. Functioning as another key cerebrovascular interface, similar to the blood–brain barrier, choroidal epithelial cells are held together by apical tight junctions which highly regulate the movement of solutes between the blood and CSF [77,78]. This dynamic flux of solutes is key in maintaining electrolytic homeostasis and transmission of ionic signaling factors. As such, there are a great variety of transporters, channels, and receptors present along the apical and basolateral surfaces of the choroid plexus which allow for complex and coordinated control of solute passage [79,80,81]. Transient receptor potential vanilloid-4 (TRPV4) is a known cation channel which plays a notable role in this ion transport, due to its ability to serve as a hub of activity and drive activation of several other transporters [82,83]. Numerous studies have illuminated the complex, paradoxical function of TRPV4 channels in inflammatory cascades involving nuclear factor kappa beta (NF-κB), tumor necrosis factor alpha (TNF-α), interleukin 1 beta (IL-1β), transforming growth factor beta 1 (TGF-β1), and interleukin 6 (IL-6) [83,84,85,86,87,88]. As such, the choroid plexus serves as the key junction between the circulation and the CNS. Because the blood–brain barrier is immature in the developing brain, so too is the choroid plexus, and thus transduction of systemic inflammation through the choroid plexus is likely heightened [81,89,90,91]. 

### 2.2. Mechanisms of Choroidal Cell Death

**Figure 2:**
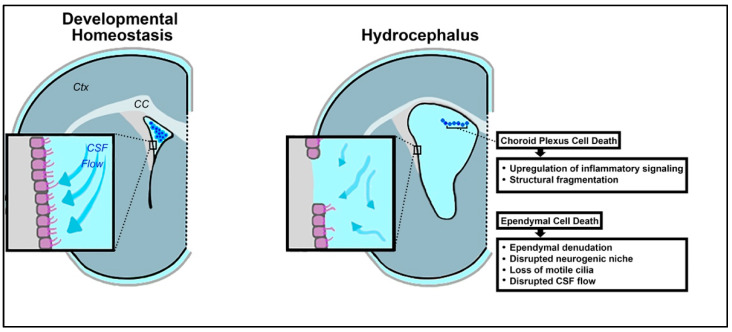
Schematic illustrating loss of developmental homeostasis in the choroid plexus and ependyma in the context of hydrocephalus. In the perinatal brain, dysregulation of the ventricular microenvironment encompasses loss of ependymal motile cilia, choroid plexus injury, and changes in the neurogenic niche. These changes are concomitant with choroid plexus cell death and ependymal cell death. (Ctx = cortex, CC = corpus callosum).

The choroid plexus exhibits upregulated activity in the setting of inflammation [92,93,94]. In fact, it has been broadly hypothesized that hyperactivity of the choroid plexus, and the resulting overproduction of CSF, contributes to impaired CSF dynamics in hydrocephalus (Figure 2) [95,96]. Cell death within the choroid plexus has also been reported, secondary to hemorrhage, infection, and neuroinflammation [97,98,99]. 

Following IVH and related ventricular dilation in a preterm rabbit pup model of IVH, upregulation of several inflammatory mRNAs has been reported in choroid plexus cells, at both 24 and 72 h post-hemorrhage (Figure 2) [100]. These transcripts include NF-κβ, IL-1β, IL-6, TNFα, toll-like receptor 4 (TLR4), Fas cell surface death receptor (FAS), interleukin 8 (IL-8), and C-C motif chemokine ligand 2 (CCL-2). In conjunction with excess pro-inflammatory mRNAs, cell death was visualized in the epithelial cells of the choroid plexus, as evidenced by structural fragmentation and caspase expression [100]. These results indicate that high levels of inflammation associated with PHHP are likely detrimental to the choroid plexus. Both apoptotic and necrotic processes have been recognized in vitro in choroidal epithelial cells as a result of oxidative stress and upregulation of inflammatory mediators [100,101]. 

Further, choroid plexus cell death was also found to result from exposure to CSF contaminated by hemorrhage-related components, including factors resulting from hemoglobin breakdown [102]. This introduces a concept known as ferroptosis, cell death associated with lipid peroxidation related to iron metabolism.

## 3. Cell Death in the Ependyma

### 3.1. Cellular Structure of the Ependyma

The ependyma is a single-celled lining present along the interior surface of the cerebral ventricular system [103,104]. This thin layer is made of ependymal cells, a supportive neuroepithelia responsible for providing a barrier between brain parenchyma and CSF, in addition to supporting the function of neural cells [3]. Ependymal cells are derived from radial glial cells, a type of embryonic neural stem cell, and develop in a gradient-like fashion from the caudal to the rostral regions of the brain during the embryonic period of neurodevelopment [74,105,106,107]. The ependymal layer is fully generated by 22 weeks’ gestation, after which the cells continue to mature through 6 months postnatally [108,109]. Notably, this corresponds to the timepoint most commonly associated with, and impacted by, preterm birth. Mature ependymal cells are post-mitotic epithelial cells that possess a severely limited capacity for additional proliferation or repair [105,106,110,111]. Ependymal cells are tightly spatially organized across the ventricular surface, forming characteristic pinwheel structures in which ependymal cells surround small apical endings of neuronal and glial progenitor cells [109,112,113,114]. Along the ventricular walls, ependymal cells are held together by desmosome junctions, forming an uninterrupted epithelial layer which allows for restricted diffusion of CSF into the CNS [115].

Of great significance, the ependymal cells which line the cerebral ventricles are multiciliated [116]. Ependymal cells can possess tufts consisting of hundreds of motile cilia that move in a coordinated whip-like motion to propel CSF across the ependymal surface [117]. During the process of differentiation from radial glial cells, centrioles assemble and anchor at the apical plasma membrane, eventually forming the basal bodies of motile cilia, which initially project from the cellular surface in disorganized orientations into the ventricular lumen [116,118]. As the maturation process progresses, the cilia grow longer and the basal bodies align in a direction according to cellular polarity and propelled fluid motion [119,120,121,122]. The subsequent metachronic beating of these cilia tufts creates regular, directed flow patterns of cerebrospinal fluid circulation near the ependymal surface within each ventricle [70,123,124,125,126,127,128]. The proper development and function of motile cilia on ependymal cells is crucial in maintaining fluid homeostasis within the CNS, and as such has critical implications in both brain development and function [129,130,131,132,133]. Defective ependymal cells involving diminished or asynchronous ciliary movements have been found in several major neurological disorders, including hydrocephalus [41,134].

In relation to infantile hydrocephalus, the synchronous beating of ependymal motile cilia is imperative to assure proper CSF circulation and prevent excess CSF accumulation under pressure [129]. Conditions that impair ependymal cilia motility can contribute to the onset of hydrocephalus [129,135,136]. Specifically, imaging of cilia in posthemorrhagic hydrocephalus model systems has provided visual evidence both of cilia loss and of the flattening or tangling of the remaining cilia [41]. Further, genetically induced ciliopathies which reduce ciliary motile function have also been found to induce hydrocephalus [121,129,135,137,138,139,140,141,142,143]. While ciliary function is of great concern in the study of hydrocephalic pathophysiology, it is imperative that the overall health of the underlying ependymal cells also be given ample consideration. Here, the damage, denudation, and death of ependymal cells in the setting of PHHP will be discussed. 

### 3.2. Mechanisms of Ependymal Cell Death

The importance of healthy and fully functional ependymal cells with their motile cilia during the embryonic and postnatal periods cannot be understated. While a loss of ependymal cells or destruction of the ependymal layer is well recognized, very few reports have investigated the path to depletion of ependymal cells. Here, the limited existing literature will be reviewed as a starting point for further investigation.

Ependymal cell death could ensue via two distinct paths in PHHP: (1) as a causative force, or (2) as an exacerbating manifestation. First, ependymal cell loss, and the concomitant loss of ependymal motile cilia, could hinder normal CSF circulation through the ventricular system, and therefore act as an initiating contributor to the onset of hydrocephalus [70,124]. Second, cell death in the ependyma could occur as a downstream effect within hydrocephalus pathology, and exacerbate a pre-existing problem [144]. Given the diverse agents, pathologies, and presentations of childhood hydrocephalus, it is quite likely that ependymal cell death occurs in either sequence of events. 

Ependymal denudation, or the process in which ependymal cells of the ventricular epithelium vacate their position on the ventricular surface, has been widely reported in cases of hydrocephalus both in model systems and in pediatric patients (Figure 2) [145,146,147,148]. While there have been some conflicting data on whether the evacuated ependymal cells are dead or simply detached, a growing amount of evidence suggests that cell death in the process of denudation is commonplace [3,146].

#### 3.2.1. Ependymal Cell Death Precipitates Hydrocephalus

There is a substantial body of research which supports the concept that ependymal death precipitates hydrocephalus, via loss of ciliary propulsion resulting in excess CSF accumulation [135,143,149]. 

On a molecular level, vascular endothelial growth factor (VEGF) is present in the CSF of humans with hydrocephalus, and causes ventriculomegaly and ependymal changes in rats [115]. In model systems with VEGF-induced hydrocephalus, E-cadherin levels have been found to be modified [115]. Interestingly, E-cadherin is replaced by N-cadherin in the process of neurulation [150]. N-cadherin is understood to play a role in the cell junctions of mature ciliated ependymal cells, and experimental blockage has been found to induce widespread apoptotic ependymal cell death [151,152]. Therefore, it is possible that N-cadherin levels may also be affected during fetal neural development via VEGF. 

Ependymal cell death via necrosis has also been reported in relation to hydrocephalus. It was found that deletion of the selective protease UBP43 in mice results in spontaneous necrosis of ependymal cells, the collapse of the cerebral aqueduct, and subsequent development of hydrocephalus [153]. In this investigation, cellular lysis and intact, uncondensed nuclear materials were identified in the lumen of the ventricle, refuting apoptosis but confirming ependymal cell necrosis [153]. 

Providing more evidence for ependymal death contribution to hydrocephalus, sorting nexus family member 27 (SNX27)-deficient mice were found to have ependymal layer defects in the form of reduced ciliary and ependymal cell density on the ventricular surface, which led to the evolution of severe postnatal hydrocephalus [149]. Similarly, mice with a knock-out of vacuolar protein sorting-associated protein 35 (VPS35), a protein known to be critical for ependymal cell survival, developed enlarged lateral ventricles and microglial activation redolent of hydrocephalus [154]. In addition, it has been found that p73 is an important molecule and its dysregulation can result in ependymal cell death and hydrocephalus [155,156,157]. p73 knockout mice exhibit ependymal cell death with the development of hydrocephalus and Afadin knockout mice develop ventricular walls which are almost totally barren due to ependymal cell detachment prior to hydrocephalus onset [155,156,157,158,159,160]. Of note, p73 is extremely versatile and is involved in multiciliogenesis and in the induction of apoptosis, while Afadin is a factor in cell-cell adhesion [161,162]. In another related study, programmed embryonic ependymal wall denudation in *hyh* hydrocephalic mutant mice was found be nearly completed prior to induction of hydrocephalus [163].

Ependymal cells are highly sensitive to infection by a variety of viral vectors [106]. This outlines a clear avenue for ependymal cell death induction in the initiation of post-infectious hydrocephalus. One study found evidence of ependymal cell necrotic death occurring as a result of intracerebral inoculation of vesicular stomatitis virus (VSV) [164]. Hydrocephalic development then followed as a result of the destruction of the ependymal layer. A review by Sarnat published in 1995 also heavily stressed that viral, non-sterile infection of the ependymal layer could lead to hydrocephalus [144]. Further, this pathway could play an initiating or exacerbating role in post-hemorrhagic hydrocephalus, where leakage of blood into the ventricular system may introduce contaminants formerly retained by the blood–brain barrier [163,165].

#### 3.2.2. Ependymal Cell Death Exacerbates Hydrocephalus 

In some cases, rather than ependymal cell death serving as the primary driver of hydrocephalus, ependymal cells undergo cell death within a broader pathological process. For example, a study characterizing a lysophosphatidic acid (LPA)-induced model of fetal-onset hydrocephalus found evidence of ependymal cell death, phagocytosis, and denudation [3]. Following fetal intracerebral administration of LPA, a blood-borne component of infection, destruction of the ciliary microtubule axis and pervasive damage to cell membrane integrity were found via transmission electron microscopy. At later timepoints, scanning electron microscopy also showed abnormal cell morphology on the ependymal surface, later identified as microglial macrophages of the innate immune system recruited to the site of ependymal cell damage [3]. Apoptosis of the ependymal cells was also noted shortly after, identified via fluorescent staining for caspase activation [3]. Similarly, significant loss of ependymal cells on the ventricular walls was also noted in swine models of hydrocephalus following intraventricular hemorrhage [166]. Overall, these findings present a compelling theory that progression from CNS insult to hydrocephalus includes primarily innate immune cell mobilization in response to damage, followed by apoptotic death of ependymal cells and denudation of the ventricular wall (Figure 2).

Ependymal cells are generally susceptible to inflammation [167,168,169,170]. Locally activated microglia have been implicated in pathological roles related to ependymal cell health and survival [154]. Ependymal cells also possess receptors which have binding affinity for specific cytokines, including C-X-C motif chemokine ligand 12 (CXCL12) and interferon alpha (INFα) [170]. Further, T-helper cells bind directly to ependymal cells via Fas-FasL binding, which could play a role in both ependymal cell dysfunction and death [171]. In hydrocephalus, an inflammatory response is activated regardless of the causative agent (e.g., hemorrhage, infection, trauma, genetics). As such, the possibility that factors essential to the persistence of inflammation could provoke ependymal cell death stands to support the idea that ependymal loss may not be a causative factor in the development of hydrocephalus, but instead may contribute to the worsening pathology as the disease progresses. 

Ependymal cell loss is greater in neonates who experience both IVH and hydrocephalus, compared to those who recover from IVH without progression to hydrocephalus [172]. Flattening or loss of the ependymal layer has been hypothesized as the result of increased intracerebroventricular pressure and ventricular stretching exerting a compressive and atrophic force directly upon the ependymal cells [144,173,174]. Specifically, histological evidence of ependymal destruction following extensive ventricular dilation exists [144,175]. The degree of ependymal cell damage and death correlates markedly with the degree of ventricular enlargement, more than with underlying etiology [144]. These data support the hypothesis that ependymal loss can result from the evolution of hydrocephalus and, therefore, can serve as an exacerbating factor in progression and persistence of hydrocephalus.

In sum, the ependymal layer normally serves a multitude of roles [144,176]. Despite this critical function, the single-celled layer is understood to be inherently quiescent and has been found to lack the ability to self-renew [177]. Therefore, it is not able to regenerate to repair itself after injury or pathogenic exposure [106]. As such, the death or loss of ependymal cells due to hydrocephalus early in development will likely have lasting effects beyond the implicit disruption of CSF dynamics. Ependymal damage is associated with abnormal neurogenesis and functional brain development especially in neonates in clinical studies [178,179]. Preventing such damage should be a high priority in future research efforts. 

### 3.3. Downstream Impacts of Ependymal Cell Loss

A critical concept to consider when evaluating the multi-fold impact of ependymal cell death includes the differentiation between cell loss resulting in damage and death, and loss resulting in consequences of impaired development. As a result of the age of onset of hydrocephalus, repercussions occur at critically sensitive timepoints in neurogenesis and neurodevelopment [180]. Therefore, regardless of the initiating factors inducing ependymal cell death, it is clear that loss of the ependyma can have widespread and long-lasting consequences on the immature CNS, in addition to those which are inherent to hydrocephalus. 

Most directly, ependymal cell death eliminates the multi-ciliated surface of the ependyma. As discussed earlier, loss of these crucial cilia and their synchronous beating pattern results in uncoordinated, unpropelled movement of CSF through the ventricular system, and excess CSF accumulation. Stagnant CSF flow may promote hydrocephalus [3,135]. While aberrant CSF circulation is undoubtedly an important outcome, loss of ependymal cells can also have significant impact on neurodevelopment.

In addition to losing cilia and inhibiting CSF pulsatility, loss of the ependyma results in the migration of reactive glia into locations previously occupied by ependymal cells [144,181,182]. Increased expression of genes involved in astrocytosis and microgliosis has also been reported, including those related to cytokine signaling and apoptotic pathways [183]. It is likely that this robust glial response serves as an exacerbating factor in the pathology of hydrocephalus, abnormal neurogenesis, and persistent inflammation.

Additionally, the fetal ependyma is thought to act as a secretory structure during neurodevelopment and plays a role in the proliferation of nearby neural progenitors [184]. Ependyma which has accumulated significant damage may not be able to adequately regulate the movement of fluid, ions, and small molecules between the CSF in the ventricular lumen and the surrounding brain parenchyma [144].

Furthermore, it has been found clinically that ependymal cell loss disrupts several underlying periventricular regions critical for advancing neurogenesis [108]. The germinal matrices found in the ventricular zone (VZ) and subventricular zone (SVZ) are crucial for brain development [108,185]. Denudation of the ependymal layer in fetuses with hydrocephalus results in immediate loss of the VZ, structural anomalies which expose the SVZ, and aberrant exodus of immature neuroblasts into the ventricular lumen [181]. The disorganization of these germinal zones has severe consequences for the renewal, maturation, and distribution of radial glial cells and other stem-like neural cell precursors [186,187]. Specifically, it has been found that neural progenitor cells can be cultured from the CSF of premature infants with hydrocephalus, indicating abnormal localization of neuroblasts [188]. Changes in cell lineages following SVZ disorganization have also been described [94]. Additionally, ependymal cilia facilitate transport of developing neural cells to their final destinations in the cerebral framework [130]. As such, ependymal cell death results in downstream damage to the germinal VZ and SVZ as well as loss of cilia which assist migration, thereby precipitating abnormal neurogenesis in addition to the evolution of hydrocephalus pathology.

## 4. Cell Death in the Glymphatic System

### 4.1. Cellular Structure of the Glymphatic System

**Figure 3:**
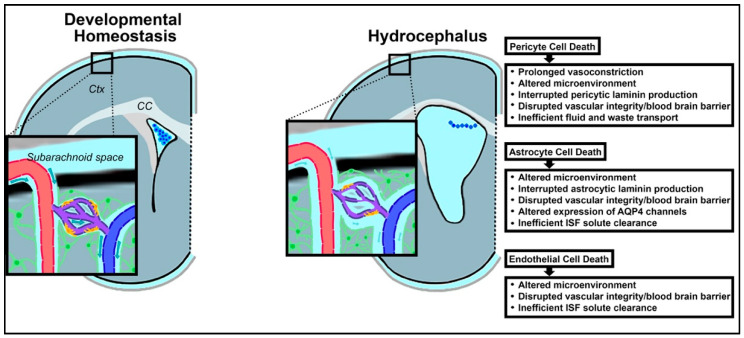
Schematic illustrating the loss of developmental homeostasis in the glymphatic system in the context of hydrocephalus. In the developing brain, dysregulation of the ventricular microenvironment also encompasses glymphatic disruption and further alterations in the neurogenic niche. These changes may be instigated by pericyte (yellow), endothelial (dark purple), and astrocytic (green) cell death.

The glymphatic system, which has recently gained wider recognition, is a waste clearance system for the CNS [189]. The CNS lacks the lymphatic drainage system found throughout the remainder of the human body. Thus, the dynamic glymphatic system fills the gap in solute and fluid elimination within the brain, and assists in maintenance of the high metabolic rate. Termed the glymphatic system as a description of its “glial lymphatic” nature, meningeal lymphatic endothelial cells (LECs) form lymphatic vessels in the dural sinuses which branch inferiorly. [190]. Below, the functional structure forms a series of perivascular channels surrounding the penetrating arterioles which branch off from the pial arteries in the subarachnoid space (Figure 3) [189,191]. These created spaces are known as Virchow–Robin spaces, and are filled with CSF [192]. They are bound on one side by the leptomeningeal cells which coat the blood vessel, and on the other by astrocytic endfeet [193]. As the vessel extends deeper into the cerebrum, it becomes continuous with the basal lamina before reaching the capillary level at the termination of the vessel. At this depth, the endothelial cells forming the vessels and the neighboring pericytes are separated from the astrocytic endfeet by the thin extracellular matrix of the basal lamina [194]. These endfeet form the boundary of the perivascular space and highly express aquaporin-4 (AQP4) [191,195,196].

Functionally, this diverse collection of cell types and unique structural architecture provide for the flow of CSF from the subarachnoid Virchow–Robin spaces down to the AQP4 channels of the astrocytic endfeet [197]. Facilitated transport into the deep brain parenchyma allows for exchange of CSF with interstitial fluid (ISF) [191]. Driven by pressure gradients and aided by the porous basal lamina and plentiful aquaporin channels, these cerebral fluids are convectively pushed towards the perivenous spaces [191]. Accumulating in this space adjacent to the deep cerebral veins, the ISF is directed superficially, out of the CNS, and eventually drains into the cervical lymphatic system [198].

In a healthy CNS, the glymphatic system clears waste and assists with fluid circulation and distribution [199]. The glymphatic system is most active during sleep, with the activity surrounding the convective flow of fluids suspended during wakefulness [200]. Disruptions to the glymphatic system may contribute to the pathogenesis of neurological diseases and disorders, either chronic or acute [201,202]. 

The glymphatic system provides a critical avenue of fluid interchange and transport in CSF dynamics and serves as a potential region for intervention for hydrocephalus. Death of the astrocytes, pericytes, and endothelial cells which comprise the neurovascular units of the glymphatic system are reviewed in the context of hydrocephalus early in life, and the impact of such loss on disease progression and subsequent neurodevelopment will be discussed (Figure 3).

### 4.2. Mechanisms of Glymphatic Cell Death

#### 4.2.1. Pericytes

Pericytes are a type of capillary-associated mural cell which wrap around the vessel-forming endothelial cells throughout the body, with the highest proportion found within the CNS [203,204,205]. In the CNS, they fortify vessel walls and interact with astrocytes as part of the blood–brain barrier (BBB) [206]. Similar to ependymal cell loss, pericytic cell death has been shown to serve both as an initiating factor in the onset of hydrocephalus, as well as an exacerbating factor in the development of hydrocephalus, specifically via phagocytosis [207,208,209].

Within the neurovascular unit of the glymphatic system, astrocytes, pericytes, and endothelial cells all produce and secrete unique isoforms of laminin in formation of the basal lamina [210,211]. Specifically, endothelial and astrocytic-derived laminins regulate vascular integrity at different points across the lifespan. Pericytic laminin plays a significant role in the development of ventricular size, as well as in constructing and maintaining the BBB [209]. In a mouse mutant with conditional knockout of pericytic laminin, BBB failure and hydrocephalus onset occurred in a significant proportion [209]. These results suggest that pericyte death, and the subsequent loss of pericytic laminin in the extracellular matrix of the glymphatic system, contributes to hydrocephalus [211], and suggests a possible causative relationship between pericyte cell death and subsequent development of hydrocephalus. 

Spontaneous excess pericyte death is highly unlikely to occur without stimuli. In PHHP, IVH could fill this role as a precipitating factor in inducing pericytic cell death [212,213,214]. In very preterm infants, IVH is thought to occur in the setting of transient hypoxia-ischemia events, which may also impact the rest of the parenchyma [215]. Pericytes have been found to be particularly vulnerable to ischemia, in addition to other microenvironmental stressors including inflammation and reactive oxygen species, and are more likely than other neural cells types to die in low-oxygen environments [216]. In fact, a lack of oxygen leads to widespread pericyte cell death and persistent pericyte contraction [216,217]. This initiates what is termed the “no-reflow phenomenon” and has been widely observed, including among patients suffering diverse types of cerebral hemorrhage [205,218,219]. With the ischemic death of pericytes in the neurovascular unit, the local capillaries become fixed in a constricted shape, prohibiting the return of blood flow and instead causing a period of prolonged vasoconstriction which worsens oxygen deprivation in the surrounding brain parenchyma [217,220]. Pericytic cell death also has been found to result in dysfunction of the glymphatic system as a whole, preventing efficient transport of fluid and waste, and further contributing to hydrocephalic fluid buildup in the brain and increased ICP (Figure 3) [221,222].

#### 4.2.2. Astrocytes

In addition to the direct impact that the loss of pericytes has on the BBB and cerebral vasculature, pericytic death has also been found to have significant impacts on the function of astrocytes, another key element of the neurovascular units which make up the glymphatic system [223]. 

Astrocytes are the most ubiquitous cell type in the CNS [224]. Like ependymal cells, astrocytes are responsible for fulfilling a variety of roles spanning from structural support of neuronal axons to directing the flow of fluid and blood [225]. In the glymphatic neurovascular unit specifically, astrocytic foot processes form a loose sheath around the cerebral vessels. In a healthy CNS, the processes are heavily lined with peri-microvessel aquaporin-4 channels which facilitate the transport of brain water, and have also been suggested to have a role in movement of ions, metabolites, and soluble proteins [195,226]. 

Astrocytes are implicated in hydrocephalus in a myriad of ways, in alignment with their multi-functionality in the CNS. There is some evidence that altered astrocyte functionality may be implicated in hydrocephalus pathophysiology, although the direct pathways have yet to be completely elucidated. One study involving G_i_-coupled Ro1 and double transgenic mice found that activating specific astrocytic G-protein-coupled receptor (GPCR) signaling pathways could initiate hydrocephalus [227]. On the contrary, reactive gliosis is common in hydrocephalus, serving as an exacerbating factor spurred on by neuronal damage as a result of trauma, infection, or ischemia, among other agents [228]. Gliosis refers to the heightened proliferation of glial cells in the CNS in nonspecific response to damage (Figure 3). Indeed, it has been found that glial fibrillary acidic protein (GFAP) RNA levels, characteristic of astrocytes, rise along with the evolution of hydrocephalus [227]. In the glymphatic system, astrocytosis occurring within the parenchymal space between the arterial and venous perivascular spaces is problematic. Rapid and widespread proliferation of astrocytes can functionally obstruct the flow and interchange of CSF and ISF [1,193]. This reduction in fluid drainage via the glymphatic system can in turn have an additive effect on impaired CSF dynamics (Figure 3). Additionally, the change in morphology that accompanies astrogliosis can redistribute the essential AQP4 channels, leading to reduced CSF flow [229]. These mechanisms reflect an increase in the number and altered morphology of astrocytes rather than a loss. Astrocytic cell death, while more rarely reported in the literature, has also been identified and could have significant implications on hydrocephalic progression [230,231]. Acknowledging the limited access to human pathological samples, astrocytic cell death amongst specific subtypes or regions may have more impact during neurodevelopment than is currently appreciated, and would benefit from future study.

Most existing reports of astrocytic cell death emphasize an ischemic environment as a critical, inciting factor [232,233,234]. As discussed previously, in hydrocephalus resulting from IVH or trauma, cerebral blood flow can be disturbed, resulting in minimal transfer of glucose and oxygen to surrounding tissues. In such a toxic microenvironment, neural cells of any type, including astrocytes, can undergo necrotic death [235]. While astrocytes are generally more resistant to stressors than neurons, upregulation of caspases has shown that subsets of astrocytes undergo apoptosis in such conditions, particularly in an immature brain [230,236]. Additional investigation involving cell cultures has also shown that astrocytes can be triggered to undergo many types of programmed cell death precipitated by cytokine dysregulation and oxidative stress [230,237]. These factors are implicated in the progression from precipitating etiology to hydrocephalus. 

After a CNS insult, in addition to astrocytic population shifts, aquaporin-4 channels are also lost. A reduction in AQP4 reduces rates of water diffusion in the CNS [238,239]. Aqp4 knockout mice exhibit increased water content in the extracellular matrices of the parenchymal space, indicating that AQP4 channel loss could impair fluid homeostasis and drainage [240,241]. In these same Aqp4 null mice, lack of AQP4 channels resulted in a ~70% increase in ISF solutes, demonstrating that both fluids and protein accumulate in the parenchyma without the proper clearance mechanisms [191]. Upregulation of AQP4 is a homeostatic countermeasure employed in the setting of hydrocephalus in order to help to disburse accumulated CSF and reduce high ICP. Specifically, elevated expression of AQP4 in astrocytic endfeet at the brain–fluid interface was detected, correlative with evolving congenital hydrocephalus in a rat model [242]. Similarly, in a rat model of communicating inflammatory hydrocephalus, the degree of AQP4 upregulation trended closely with the volume of CSF present, providing evidence that upregulation of aquaporin channels in astrocytic endfeet may occur to counteract excess CSF and increased ICP [243]. While human studies are more limited, it was also found that AQP4 protein levels in CSF were elevated in communicating hydrocephalus samples compared to controls [244]. 

Without the ability to self-correct in this way, a glymphatic system lacking appropriate astrocytes is unable to drain CSF and ISF as necessary, and has been implicated in the progression of hydrocephalus. Further, astrocytic death will also have negative impacts on the structure of the extracellular matrix itself. Without astrocytic-produced laminin, the basement membrane responsible for the BBB is weakened [245]. Additional hemorrhage can result from such structural defects, which can in turn further damage the cells making up the glymphatic neurovascular unit [246].

#### 4.2.3. Endothelial Cells

Endothelial cells constitute the cerebral vasculature and are responsible for physically forming the first layer of the BBB [247]. Joined together by tight junctions which prevent the passage of any fluid or solute, endothelial cells form the innermost layer of the vessels responsible for transporting blood throughout the brain, and therefore come into direct contact with blood components [248,249]. 

As with the other cellular components of the glymphatic neurovascular unit, hemorrhage and trauma can create a toxic microenvironment and cause endothelial cell death [250]. Further, since endothelial cells are located directly at the site of extravasation, they die first [251]. Specifically, apoptosis was identified in 10% of local endothelial cells within 10 min after hemorrhage, demonstrated by increased quantities of anti-cleaved caspase-3 positive cells colocalized with the endothelial marker RECA-1 [252]. Vascular endothelial cells are directly affected by the increased ICP characteristic of hydrocephalus [242]. Additionally, complement activation in the setting of inflammation has also been shown to result in formation of the membrane attack complex, which can form holes in cell membranes and lead to endothelial cell lysis and death following hemorrhage [253,254].

Such a sizeable loss of endothelial cells affects the glymphatic system as a whole. Endothelial laminin, laminin-alpha4, is essential in the embryonic and neonatal stages of development [255]. In the setting of PHHP, hemorrhage-induced loss of endothelial cells results in underproduction of this endothelial laminin at a critical timepoint in the development of neurovasculature, just when it is needed to promote the organization and integrity of basement membranes surrounding newly generated microvessels. As such, the basement membranes of the glymphatic system can become destabilized, and may contribute to faulty drainage of cerebral fluids and wastes, exacerbating the propagation of hydrocephalus (Figure 3) [256,257].

## 5. Cell Death via Ferroptosis

### 5.1. Ferroptosis Described 

Hemoglobin (Hgb) is a carrier molecule found in blood circulation which transports both oxygen and carbon dioxide to sustain respiration [258]. Structurally, it is made up of four subunits, each with its own polypeptide globin chain and a heme group, which contains ferrous iron atoms for binding cargo [259]. Ferroptosis refers to a form of programmed cell death involving lipid peroxidation which is iron-dependent [260,261]. Unique in signaling pathways and cell morphology from apoptosis, necrosis, and autophagy, ferroptosis occurs due to Fenton reaction-driven accumulation of lipid-based reactive oxygen species (ROS) [262]. This type of cell death can be identified in cells with an intact cell membrane and nuclear structure, but with abnormal changes to the mitochondrial membrane, including outer membrane rupture, shrinkage, increased density, and loss of cristae [263,264]. Alteration of lipid peroxidation appears to have numerous genetic mediators, and can be induced by a growing number of agents, including erastin, glutamate, RSL3, DPI7, FIN56, and FIN02 [260,261,265].

### 5.2. The Role of Ferroptosis in PHHP

Aberrant levels of iron in the body can have significant effects on normal physiology and function [266]. Ferroptosis has been previously shown to play a role in a variety of tumor-related diseases, kidney injury, and neurological diseases [267,268,269,270]. Specifically, in the setting of PHHP, the hemoglobin-associated hemes found in blood are degraded by heme-oxygenase to produce free iron [263]. This has been clinically confirmed by findings of higher unbound iron in neonates with post-hemorrhagic ventricular dilation compared to neonates without [271]. The toxic microenvironment created by an intraventricular hemorrhage, germinal matrix hemorrhage or periventricular hemorrhagic infarction results in iron deposition directly into tissues. This increase in the level of iron present has the potential to induce ferroptosis in neighboring cells [272]. 

Experimentally, intraventricular injection of iron results in damage to the ependyma and ventricular enlargement, as does injection of lysed red blood cells [273]. IVH in rats also results in increases in free iron, iron-associated proteins, heme-oxygenase 1, and ferritin [274]. In one model, lateral ventricular dilation was identified within 24 h of hemorrhage [94]. Additionally, glutathione peroxide 4 (GPX4), a developmentally regulated enzyme associated with mitigating BBB damage, oxidative stress, and inflammation, is drastically reduced by increased iron concentrations [275]. The role of free iron ions in IVH-induced hydrocephalus may be closely interwoven with the inflammatory response [102,276]. In addition, in instances involving traumatic brain injury such as post-traumatic hydrocephalus, upregulation of genes involved in ferroptosis and ROS accumulation occur biologically secondary to the initial TBI [277].

A great majority of the literature describing ferroptosis in PHHP is focused on ferroptosis-driven neuronal and white matter cell death [278,279]. Ferroptosis likely also directly impacts cells of the choroid plexus, ependyma, and glymphatic system. Furthermore, ferroptosis has been implicated in glial cell death [280]. As such, investigation into ferroptosis-mediated ependymal, glymphatic, and choroidal cell death will benefit from further research.

## 6. Cell Death in White Matter

White matter in the CNS refers to the myelin-coated axonal projections which are found in the deeper, subcortical tissues of the brain [281,282]. Myelin wrapping, via oligodendrocytes, enables saltatory conduction and nerve impulse propagation, in addition to providing metabolic support to axons [283,284,285]. Cell death in white matter has been more widely studied than cell death in other cerebral regions in the setting of hydrocephalus [286,287]. Diverse forms of encephalopathy can have adverse effects on white matter, and trigger cell death mechanisms spanning from apoptosis and necrosis, to autophagy and even ferroptosis [231,288,289,290]. Cell death in white matter is generally thought to be due to a destructive outcome of hydrocephalus, rather than as an exacerbating factor that contributes the progression of hydrocephalus. 

## 7. Cell Death in Other Neural Components

Similar to white matter, cell death in other neural components within the developing nervous system occurs. Of note, transient neonatal neuronal populations, specifically the GABAergic neurons, are particularly vulnerable to the toxic microenvironmental conditions which can be induced following IVH [291]. These late migrating neurons have been found to have increased levels of apoptosis in the cerebral tissue of premature infants with perinatal brain injury and white matter lesions [292]. As GABAergic subpopulations contribute to the formation of the networks of cortex and thalamus, loss due to early cell death may result in underdevelopment or disorganization of the cortical and thalamic regions [282]. 

## 8. Discussion of Future Directions

The physiological structures, tissue organization, and causative mechanisms discussed in this review summarize the literature on cell death in the setting of PHHP. Inflammation and iron-related metabolites likely contribute to alterations of CSF dynamics via the choroid plexus, ependyma, and glymphatic system.

In addition to further refining initiating causes of CSF overproduction by the choroid plexus, cell death in this important organ should also be investigated to elucidate downstream consequences, both in the initiation and progression of hydrocephalus, as well as possible lifelong impacts resulting from loss of choroid plexus tissue. The choroid plexus secretes multiple trophic factors critical for both neurodevelopment and maintenance of the healthy mature CNS [293,294]. 

With respect to the ependyma, deeper investigation into ependymal cell death is necessary. A more comprehensive understanding of the mechanisms involved in the denudation of the ventricular wall could lead to prevention of ependymal cell and associated motile cilia loss. Preservation of proper, coordinated ciliary movement could pave the way towards the rescue of abnormal CSF flow patterns and promotion of CSF drainage, helping to halt the evolution of hydrocephalic pathology after neural injury or inflammation. 

Changes in the glymphatic system after injury require extensive investigation. Improved understanding of fluid exchange pathways and signaling involved in regulation is needed. Additionally, mechanisms of cell death occurring in each component of the neurovascular unit—pericytes, astrocytes, and endothelial cells—require further characterization to identify new targets for intervention. Another key line of investigation is to identify changes that may be prompted simply by excess CSF pressure, rather than by the precipitating insult. It is quite likely that alterations in glymphatic function may play a pathophysiological role in numerous neurodevelopmental and neurological diseases and disorders as a result of the eminent roles of the blood–brain and CSF–blood interfaces in fluid and signaling homeostasis [295,296,297].

Finally, investigation into elucidating the critical signaling pathways associated with ferroptosis as a mechanism of cell death are of paramount importance. Ferroptosis likely contributes not only to white matter injury and neuronal loss in the cortex and deep gray matter, but also to loss of neural cells which regulate CSF dynamics, including microglia, astrocytes, and the specialized cells that comprise the choroid plexus, ependyma, and glymphatic system.

As research on hydrocephalus advances, developing an improved, more comprehensive understanding of cell death as an exacerbating factor in pathogenesis is imperative. This review focuses on the context of PHHP, however many of the pathways and factors discussed are also plausible in other forms of infantile hydrocephalus as well. As such, we propose that there exists unifying pathophysiology of both inflammation-related and iron-related cell death across many forms of infantile hydrocephalus, independent of the initiating insult.

Cell death represents a permanent, and often irreversible, loss of vital components within a rapidly developing neurological system. As an effect of hydrocephalus, systemic inflammation and loss of integral cellular components of the CNS serve as pivotally destructive insults in neonates. On a broader scale, continued studies to achieve a better understanding of all diseases falling under the umbrella of encephalopathy of prematurity are of paramount importance in the mission to reverse, and even prevent, perinatal brain injury in millions of infants around the globe.
